# Regulation of extracellular serotonin levels and brain-derived neurotrophic factor in rats with high and low exploratory activity

**DOI:** 10.1016/j.brainres.2007.11.041

**Published:** 2008-02-15

**Authors:** Tanel Mällo, Kadri Kõiv, Indrek Koppel, Karita Raudkivi, Ain Uustare, Ago Rinken, Tõnis Timmusk, Jaanus Harro

**Affiliations:** a Department of Psychology, Centre of Behavioural and Health Sciences, University of Tartu, Tiigi 78, 50410 Tartu, Estonia; b Department of Gene Technology, Tallinn University of Technology, Akadeemia tee 15, Tallinn 19086, Estonia; c Department of Organic and Bioorganic Chemistry, University of Tartu, Jakobi 2, 51014 Tartu, Estonia

**Keywords:** 5-HT, serotonin, LE, low exploratory activity, HE, high exploratory activity, PFC, prefrontal cortex, DG, dentate gyrus, HI, hippocampus, PCA, parachloroamphetamine, 5-HTT, serotonin transporter, BDNF, brain-derived neurotrophic factor, NGF, nerve growth factor, AP, anterior–posterior, ML, medial–lateral, DV, dorsal–ventral, Exploratory behaviour, Anxiety, Individual differences, Medial prefrontal cortex, Hippocampus, Serotonin transporter, Neurotrophins

## Abstract

Serotonin (5-HT) system has a significant role in anxiety- and depression-related states and may be influenced by brain-derived neurotrophic factor (BDNF). This study examined extracellular 5-HT levels and expression of BDNF in rats with persistently low or high levels of exploratory activity (LE and HE, respectively). Baseline extracellular levels of 5-HT as assessed by in vivo microdialysis in conscious animals were similar in both groups in medial prefrontal cortex (PFC) and dentate gyrus (DG). No differences were found in parachloroamphetamine-induced 5-HT release in either region. However, LE animals had significantly higher levels of 5-HT transporter (5-HTT) binding in PFC and a larger increase in extracellular 5-HT levels after administration of citalopram (1 μM) into this area by retrograde dialysis. No difference in 5-HTT levels was found in hippocampus, while perfusion with citalopram was accompanied by a greater increase in extracellular 5-HT in the HE group in this brain region. LE-rats had higher levels of BDNF mRNA in the PFC but not hippocampus. In contrast, levels of nerve growth factor mRNA were similar in these brain regions of LE- and HE-rats. The differential regulation of 5-HT-ergic system in LE- and HE-rats in PFC and hippocampus may form the basis for their distinct anxiety-related behaviours.

## 1 Introduction

The serotonergic system has been implicated in the etiology of human affective and anxiety disorders and is a major target in the treatment of these conditions (Morilak and Frazer, 2004). It has also been proven to play a role in anxiety and emotional reactivity in animals and in corresponding animal models of human disorders (for a review, see Griebel, 1995). Nevertheless, serotonin (5-HT) pathways do not have a unitary function in modulating anxiety. In general, the influence of 5-HT neurons on forebrain centers has been suggested to facilitate threat-related information processing, while the 5-HT-ergic influence on brainstem may be largely inhibitory (Handley, 1995). 5-HT-ergic pathways innervating such brain regions as the frontal cortex, amygdala, hypothalamus and hippocampus have been found to be activated by anxiogenic stimuli, including psychosocial stress, conditioned fear and conflict procedures (for a review see Millan, 2003). The relationship between 5-HT-ergic activity and the affective state of the test animal is not always straightforward, as it has also been shown that an increase in 5-HT-ergic activity in different forebrain regions may be linked simply to general behavioural activity but not to specific actions or affective states (Rueter and Jacobs, 1996). Both reduced as well as increased 5-HT-ergic neurotransmission have been associated with negative emotionality (see Tõnissaar et al., 2004, and references therein).

Animals bred or preselected based on anxiety-related behaviour in one test often but not always display consistently different behavioural patterns in other tests used in anxiety and depression research (Kabbaj et al., 2000; Ho et al., 2002; Mällo et al., 2007a). Differences in the expression of anxiety are accompanied by differences in neurochemistry of amino acids, monoamines and neuropeptides (Rägo et al., 1988; Harro et al., 1990; Landgraf, 2005; Singewald, 2007). Particular interest has been paid to the 5-HT-ergic function in medial prefrontal cortex (PFC) and hippocampus (Pollier et al., 2000; Giorgi et al., 2003; Keck et al., 2005), but the results have remained rather inconclusive, possibly due to large variation in behavioural paradigms and methods used and the single variables measured. In our laboratory, we have used a test of exploratory behaviour (Otter et al., 1997) which usually yields a rather dichotomous distribution of rats into groups with low and high exploratory activity (LE and HE, respectively) with high reliability to predict further novelty-related activities (see Mällo et al., 2007a). We have shown that these two groups display, respectively, high and low levels of anxiety in several other behavioural tests and respond differently to certain pharmacological manipulations. For example, treatment with the neurotoxin DSP-4 which is selective to the locus coeruleus noradrenergic projections had different effects on spontaneous, amphetamine-stimulated (Alttoa et al., 2005) and amphetamine-sensitized (Alttoa et al., 2007) behaviour in LE- and HE-rats. DSP-4 treatment also decreased the ex vivo 5-hydroxyindoleacedic acid levels in the nucleus accumbens and striatum in LE-rats only. We have also found that the HE-rats have higher extracellular dopamine levels in the striatum in baseline conditions as well as in response to amphetamine administration, while no difference was detected in the nucleus accumbens (Mällo et al., 2007a). In the light of the importance of 5-HT-ergic system in the anxiety- and depression-related conditions we have studied the LE- and HE-rats regarding their 5-HT extracellular levels in PFC and hippocampus (in case of microdialysis studies the target was more specifically dentate gyrus (DG)) in baseline conditions, after systemic administration of the 5-HT-releasing agent parachloroamphetamine (PCA) and after local infusion of 5-HT reuptake inhibitor citalopram. Also, 5-HT transporter (5-HTT) levels were measured in the same brain regions by radioligand binding.

Neurotrophins, including nerve growth factor (NGF), brain-derived neurotrophic factor (BDNF), neurotrophin-3 and neurotrophin-4, are a family of secreted growth factors that promote the survival, differentiation and maintenance of specific neuronal populations and regulate activity-dependent synaptic plasticity (reviewed in Bibel and Barde, 2000). Decreases of BDNF levels are accompanied by and are believed to lead to several pathologies, including neuropsychiatric disorders like anxiety-related behaviours, depression, bipolar disorder, schizophrenia and neurodegenerative disorders like Huntington's, Parkinson's and Alzheimer's diseases (reviewed in Binder and Scharfman, 2004; Angelucci et al., 2005; Duman and Monteggia, 2006). Because the promotion of the function and growth of 5-HT-ergic neurons by BDNF (Mamounas et al., 1995; Altar, 1999), as well as changes in BDNF function in affective disorders (Licinio and Wong, 2002) have been described, we also measured the levels of BDNF expression in these brain regions in LE- and HE-rats.

## 2 Results

### 2.1 Baseline extracellular 5-HT levels in LE- and HE-rats, and the effect of PCA

**Fig. 1:**
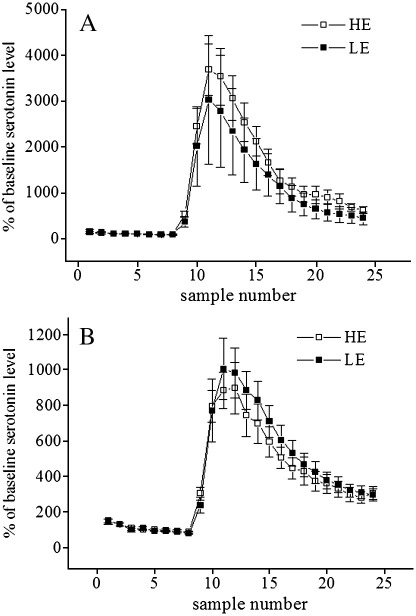
Extracellular serotonin levels in the medial prefrontal cortex (A) and dentate gyrus (B) of LE- and HE-rats after parachloroamphetamine (PCA) treatment. Samples were collected every 15 min and are presented as percentage of baseline levels (mean of samples 4.–6.). PCA (2 mg/kg, intraperitoneally) was administered before the collection of sample 8. LE – low exploratory activity rats (filled squares, *n* = 4 and 9 in PFC and HI, respectively); HE – high exploratory activity rats (open squares, *n* = 6 and 10 in PFC and HI, respectively).

In the baseline conditions, the average (mean ± SEM of samples 4.–6.) extracellular levels of 5-HT were 10.2 ± 3.4 fmol/22.5 μl sample in the LE-rats and 5.8 ± 1.1 fmol/22.5 μl sample in the HE-rats in PFC and 1.95 ± 0.27 fmol/22.5 μl sample in the LE rats and 1.97 ± 0.34 fmol/22.5 μl sample in the HE-rats in DG. Repeated measures ANOVA revealed no difference between LE and HE groups in baseline 5-HT levels in either region. The increase in extracellular 5-HT availability after administration of PCA (2 mg/kg) was also similar in both regions in both groups (Fig. 1).

### 2.2 5-HTT levels in LE- and HE-rats

**Fig. 2:**
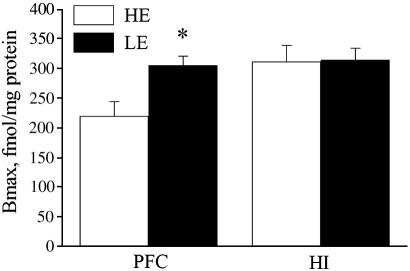
5-HTT levels measured with [^3^H]citalopram binding in membranes of the medial prefrontal cortex (PFC) and hippocampus (HI) of LE- and HE-rats. ^∗^*P* < 0.05 compared to the HE group. LE – low exploratory activity rats (filled bars, *n* = 6 in both regions); HE – high exploratory activity rats (open bars, *n* = 6 and 7 in PFC and HI, respectively).

In PFC, a significant difference between the groups in 5-HTT binding (*t* = − 2.97; *P* < 0.05) was found, with the transporter levels significantly higher in the LE group (Fig. 2). In the hippocampus, no difference was detected in 5-HTT levels between the two groups.

### 2.3 Effect of citalopram administration on extracellular 5-HT availability in LE- and HE-rats

**Fig. 3:**
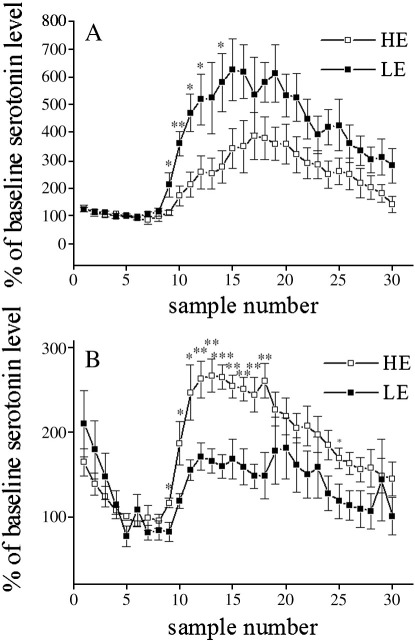
Extracellular serotonin levels in the medial prefrontal cortex (A) and dentate gyrus (B) of LE- and HE-rats, after local infusion of citalopram. Samples were collected every 15 min and are presented as percentage of baseline levels (mean of samples 4.–6.). Infusion with 1 μM solution of citalopram was made during the collection of samples 7.–16. ^∗^*P* < 0.05; ^∗∗^*P* < 0.01; ^∗∗∗^*P* < 0.0001 difference between LE and HE groups. LE – low exploratory activity rats (filled squares, *n* = 6 in both regions); HE – high exploratory activity rats (open squares, *n* = 6 and 7 in PFC and HI, respectively).

In the baseline conditions, the average (mean ± SEM) extracellular levels of 5-HT in PFC were 6.95 ± 2.16 fmol/22.5 μl sample in the LE-rats and 5.02 ± 0.55 fmol/22.5 μl sample in the HE-rats, and in DG, 9.71 ± 2.33 fmol/22.5 μl sample in the LE-rats and 10.81 ± 2.22 fmol/22.5 μl sample in the HE-rats. There were no significant differences in extracellular 5-HT levels in either PFC or DG in baseline conditions. A significant difference between LE and HE groups was found in both PFC [*F*(1,10) = 5.30; *P* < 0.05] and DG [*F*(1,10) = 6.44; *P* < 0.05] (Figs. 3A and B, respectively), when citalopram (1 μM) was administered via reverse dialysis. In PFC, the increase in extracellular 5-HT levels after citalopram infusion was about twofold higher in the LE-rats as compared to that of the HE-rats. Conversely, in DG, the increase in 5-HT levels after citalopram infusion was about two times higher in the HE-rats than the LE-rats.

### 2.4 Neurotrophin mRNA levels in LE- and HE-rats

**Fig. 4:**
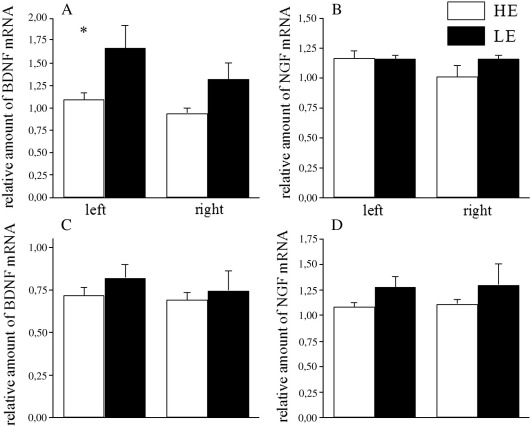
Relative levels of brain-derived neurotrophic factor (BDNF) and nerve growth factor (NGF) mRNA in prefrontal cortex (PFC) and hippocampus (HI) of LE- and HE-rats separately in left and right hemisphere: (A) BDNF in PFC; (B) NGF in PFC; (C) BDNF in HI; (D) NGF in HI. BDNF and NGF mRNA were normalized to β-actin. ^∗^*P* < 0.05 difference compared to the respective LE group. LE – low exploratory activity rats (filled bars, *n* = 5–6); HE – high exploratory activity rats (open bars, *n* = 5–6).

In the PFC, a significant effect of Exploration was revealed on the BDNF mRNA levels normalized with β-actin [*F*(1,18) = 8.48; *P* < 0.01] that were higher in the LE group in the left hemisphere; there was no exploration × left/right hemisphere interaction in ANOVA and indeed a similar difference between the LE and HE groups in the right hemisphere which missed the conventional level of significance was also notable (Fig. 4). No differences in BDNF mRNA levels were found between LE and HE animals in hippocampus. Also, no differences in NGF mRNA levels were found between LE and HE animals in either brain region.

## 3 Discussion

The present results demonstrate that there are differences in the 5-HT-ergic system in frontal cortex and hippocampus between rats preselected in the exploration box test for low and high exploratory activity. Both of these regions have been acknowledged to play significant roles in affective states in humans and are targets for treatments of mood disorders. The PFC has widespread influences on multiple components of forebrain circuits regulating anxiety states and anxiety-related behaviour. The hippocampal 5-HT-ergic system has been acknowledged to mediate an anxiogenic response (for an example, see File et al., 2000), and dysfunction in hippocampal region, especially in DG, has been ascribed an important role in stress reactions (Hindmarch, 2002).

In the present experiments, the baseline levels of 5-HT release in PFC and DG were similar in LE- and HE-rats, and no differences were detected in PCA-induced depolarization-independent 5-HT release in these regions. Previously, we have studied tissue levels of 5-HT and its metabolite in these regions and found no differences between the two groups either (unpublished data). Nevertheless, after local infusion with citalopram, the increase in extracellular 5-HT levels in LE group was greater in the PFC and smaller in DG. It is possible that the firing rate of the 5-HT-ergic projections to the PFC is higher in LE-rats, resulting in higher extracellular 5-HT levels after blockade of 5-HTT, while at baseline conditions the greater release of 5-HT is balanced by the increased re-uptake in the LE-rats that we found to have higher levels of 5-HTT in this region. Similarly, in studies on patients with bipolar disorder, 5-HTT levels have been found to be increased in medial prefrontal cortex, but not in hippocampus (Cannon et al., 2006).

Giorgi et al. (2003) have previously reported higher levels of [^3^H]-citalopram binding to 5-HT reuptake sites and greater increase in 5-HT availability elicited by local application of chlorimipramine and fluoxetine in the frontoparietal cortex in Roman high-avoidance rats, that also have been found to be more anxious in the elevated plus-maze and light/dark compartment test (Chaouloff et al., 1994), while the release of 5-HT was similar at baseline conditions with the low-avoidance (LHA) rats. Parenthetically, it should be noted that the above mentioned high-avoidance rats are also more susceptible to drugs of abuse than the LHA rats (Giorgi et al., 2007), and this may also be the case for our LE-rats which develop sensitization to a low dose of amphetamine that did not elicit this behavioural change in HE-rats (Alttoa et al., 2007). Petty et al. (1994) have shown that after exposure of rats to tail-shock stress, 5-HT release in the medial frontal cortex correlates significantly with subsequent helpless behaviour, although the basal 5-HT levels did not. It therefore seems that the increased reuptake of 5-HT in prefrontal cortical areas may develop in anxious subjects to reduce behavioural consequences of aversive stimuli.

Kulikov et al. (1997) have reported no difference in 5-HTT levels in hippocampus between Lewis and spontaneously hypertensive rats, of which the former is more anxious in the elevated plus-maze test. However, the Wistar-Kyoto rats, considered as more anxious, have been reported to have lower levels of 5-HTT binding sites in the hippocampus when compared with the less anxious spontaneously hypertensive rats (Pollier et al., 2000), and animals that are bred for high anxiety-related behaviour in the elevated plus-maze show an increased number of 5-HTT binding sites in the hippocampus (Keck et al., 2005). In the context of these controversial findings, the current results about no difference in hippocampal 5-HTT levels between LE- and HE-animals suggest that although the 5-HTT in this brain region may have a role in the regulation of anxiety-related aspects of behaviour, its effects are regulated by factors possibly not controlled for in these experiments. It certainly has to be noted that in the current experiments, 5-HTT levels were measured in the whole hippocampus of animals not subjected to microdialysis, while 5-HT availability in the presence of citalopram was measured by microdialysis in the DG only. Nevertheless, a possible mechanism for increased 5-HT levels in this region in HE-rats after local infusion of citalopram may be through differential activity of 5-HT_1B_ autoreceptors, which regulate the release of 5-HT by inhibitory feedback and have been hypothesized to be supersensitive in depression and anxiety (Moreta and Briley, 2000). The 5-HT_1B_ autoreceptor activity has previously been found to be different in PFC and DG (Adell et al., 2001), and indeed in our experiments the effect of local administration of citalopram on 5-HT levels was much smaller in DG than in the PFC which may suggest higher degree of local regulation in this brain region.

It has been previously proposed that 5-HTT-mediated increases in 5-HT-ergic neurotransmission upregulate BDNF expression via increases in cAMP response element binding protein phosphorylation (Duman et al., 1997, 1999; Nibuya et al., 1996), but it has also been demonstrated that male and female mice with partial (5-HTT +/−) or complete (5-HTT −/−) reductions in 5-HTT expression have no differences in BDNF protein levels in frontal cortex and striatum, as compared to 5-HTT +/+ mice (Szapacs et al., 2004), suggestive that at least the regions studied lack 5-HTT-mediated control over this neurotrophin system. Apparently, the decreased BDNF levels are accompanied by rather subtle changes in the 5-HT system that may not be significant at baseline conditions (i.e. in microdialysis studies), but which nevertheless become substantial in more challenging conditions. For an example, when BDNF +/− mice were interbred with 5-HTT knock-out mice, the lack of either gene on its own had no significant effect on behaviour in the elevated plus-maze test, but the combined effects of partially or totally knocking out both genes significantly decreased activity on the open arms (Ren-Patterson et al., 2006). In the present experiments, the levels of BDNF mRNA were found to be higher in the PFC of the LE-rats as compared to the HE-rats, suggesting that BDNF gene expression is increased on the level of transcription or mRNA stability. NGF mRNA levels were found not to be different in these two groups. This, combined with the current results from the microdialysis study in PFC, fits with the findings of BDNF going hand in hand with increased 5-HT availability (Altar, 1999), possibly through a 5-HTT-controlled process. Nevertheless, it should be acknowledged that our results are not indicative of any direct association between BDNF and 5-HTT expression.

The results contradict with the common BDNF hypothesis of depression that predicts decreased levels of BDNF mRNA in depression-related brain areas (Licinio and Wong, 2002). However, this view is already questioned, as bulbectomized mice that have depressed-like phenotype have recently been found to have significantly increased levels of BDNF protein in hippocampus and frontal cortex (Hellweg et al., 2007). Also, in two rat models of depression-like states, no differences as compared to controls or even an upregulation of BDNF has been found in several brain areas (Angelucci et al., 2004; Vollmayr et al., 2001). These results suggest that the role of this neurotrophic factor in mood disorders is more complex than originally thought. For example, the possibility has to be considered that the change in BDNF levels in rodent models of anxiety and depression might indicate an attempt towards neurochemical adaptation that remains unsuccessful in behavioural regulation.

The current results suggest that the persistently different profiles of anxiety-related behaviour can be related to the differences in the functional qualities of the 5-HT systems in prefrontal cortex and hippocampus. The increased BDNF mRNA levels in HE-rats may be influencing or elicited by these differences between the groups.

## 4 Experimental procedures

### 4.1 Animals

In all experiments, male Wistar rats (Scanbur BK AB, Sweden) were housed in groups of four in standard transparent polypropylene cages under controlled light cycle (lights on from 08:00 h to 20:00 h, the lighting in the animal room was 320–400 lx, depending on the height of the shelf on which the cage was located) and temperature (19–21 °C), with free access to tap water and food pellets (diet R70, Lactamin, Sweden). In the microdialysis experiment, only data from the animals with correct probe location are presented. The experimental protocol was approved by the Ethics Committee of the University of Tartu.

### 4.2 Exploration box test

The exploration box was carried out as described in Mällo et al. (2007a). The rats were divided into LE and HE activity groups based on the median value of the sum of exploratory activity during the second testing. The average scores of exploratory activity varied between 0 – 6 and 92 – 178 activity units for LE and HE animals, respectively, over all experiments.

### 4.3 Microdialysis

Twenty-five LE-rats and 29 HE-rats were subjected to the microdialysis procedure. The microdialysis probe was inserted into the left hemisphere PFC in 10 LE and 12 HE animals and into the left hemisphere DG in 15 LE and 17 HE animals. Two animals were studied daily over a period of four weeks following the second exploration box test. The animals were anaesthetized with chloral hydrate (350 mg/kg intraperitoneally) and mounted in a Kopf stereotactic frame. A self-made concentric Y-shaped microdialysis probe with 5 mm shaft length and active membrane on the whole length was implanted into the PFC according to the following coordinates: AP, + 3.3; ML, + 0.8; DV, − 5.0, according to Paxinos and Watson (1986). A probe with 4 mm shaft length and 1 mm active tip was implanted into DG with the following coordinates: AP, − 4.3; ML, + 2.2; DV, − 3.8. The dialysis membrane used was polyacrylonitrile/sodium methalyl sulphonate copolymer [Filtral 12; inner diameter (ID), 0.22 mm; outer diameter, 0.31 mm; AN 69, Hospal, Bologna, Italy]. Two stainless steel screws and dental cement was used to fix the probe to the scull. After the surgery, rats were placed in 21 × 36 × 18 cm individual cages in which they remained throughout the rest of the experiment. Rats were given about 24 h for recovery and microdialysis procedure was conducted in awake and freely moving animals as described in Mällo et al. (2007a). We have not detected any significant effects of the surgery and probe implantation on the animals' behaviour in our previous studies. In short, after connecting the animal to the microdialysis system, the perfusate was discarded during the first 60 min to allow stabilization. Then 6 or 7 samples were collected for analysis, followed by either intraperitoneal injection of PCA (2 mg/kg, in 4 LE and 6 HE animals with microdialysis probe in the PFC and in 9 LE and 10 HE animals with microdialysis probe in the DG), or local administration of citalopram (1 μM) by reverse dialysis for 2.5 h (in the PFC of 6 LE and 6 HE animals and in the DG of 6 LE and 7 HE animals). Following the PCA injection, another 18 samples were collected; 14 samples were collected after the cessation of citalopram administration. The samples were collected in 15-min periods directly into a 50-μl loop of the electrically actuated injector (Cheminert C2V, Vici AG International, Switzerland) and injected automatically into the column in order to determine the quantity of 5-HT in the samples online by using HPLC with electrochemical detection.

Upon completion of the experiment the animals were deeply anesthetized with chloral hydrate (350 mg/kg, intraperitoneally) and decapitated; the brains were removed, immediately frozen and kept at − 80 °C. The brains were sectioned on a cryostatic microtome (Microm GmbH, Germany) and probe placements were determined according to the atlas of Paxinos and Watson (1986).

### 4.4 Radioligand binding

Samples from the frontal cortex of 6 LE-rats and 6 HE-rats, and from the hippocampi of 6 LE-rats and 7 HE-rats were collected for the determination of 5-HTT levels by specific binding of [*N*-*methyl*-^3^H]citalopram as described in Mällo et al. (2007b). The PFC samples were collected from the right hemisphere (contralateral to the microdialysis site) of the frozen brains of the animals that had undergone the microdialysis experiment immediately before the probe localization determination, while the DG samples were collected from the right hemisphere of naive animals that were decapitated and brains immediately dissected on ice.

### 4.5 RNA isolation, cDNA synthesis and quantitative real-time PCR

Brain regions of six LE-rats and six HE-rats, not used in any experiments but for the selection, were dissected and used for total RNA isolation and cDNA synthesis as described previously (Pruunsild et al., 2007). Levels of total BDNF and NGF mRNA were quantified with qPCR Core kit for SYBR® Green I No ROX (RT-SN10-05NR, Eurogentec, Belgium). All reactions were performed on LightCycler 2.0 thermocycler (Roche) using the following temperature cycling conditions: 10 min at 95 °C (initial denaturation step), then 45 cycles of 5 s at 95 °C, 10 s at 55 °C and 10 s at 72 °C. All PCR reactions were performed in triplicate and normalized to β-actin (ACTB) mRNA levels. The following primers were used: BDNF_cod_s GGCCCAACGAAGAAAACCAT, BDNF_cod_as AGCATCACCCGGGAAGTGT, NGF_s TTGCCAAGGACGCAGCTTTCTA, NGF as CAACATGGACATTACGCTATGCA, ACTB_s ATGGAATCCTGTGGCATCCAT and ACTB_as CCACCAGACAGCACTGTGTTG.

### 4.6 Data analysis

Results of the microdialysis experiments are expressed as a percentage of basal 5-HT values. Basal 5-HT values were calculated for every individual animal as the mean of the last three consecutive baseline samples before the start of citalopram or PCA administration. The data were analysed with repeated measures ANOVA. Data of the 5-HTT binding were analysed by means of non-linear least squares regression using a commercial program GraphPad PRISM™ (GraphPad, San Diego, CA, USA) and obtained parameters compared with *t*-tests. Q-PCR data are expressed as BDNF or NGF mRNA levels relative to the reference β-actin mRNA levels, with the expression level of 1.0 for a randomly selected sample, and analysed with a two-factor (exploration × left/right hemisphere) ANOVA. When appropriate, post-hoc comparisons were made with the Fisher's PLSD test. Statistical significance was set at *P* < 0.05 in all analyses. All statistics were made using StatView 5.0 software (SAS Institute Inc., Cary, NC, USA).
